# The ventral peptidergic system of the adult ascidian *Ciona robusta* (*Ciona intestinalis* Type A) insights from a transgenic animal model

**DOI:** 10.1038/s41598-020-58884-w

**Published:** 2020-02-05

**Authors:** Tomohiro Osugi, Yasunori Sasakura, Honoo Satake

**Affiliations:** 1 Bioorganic Research Institute, Suntory Foundation for Life Sciences, Kyoto, Japan; 2 Shimoda Marine Research Center, University of Tsukuba, Shimoda, Japan

**Keywords:** Morphogenesis, Cell biology, Evolution, Neuroscience, Endocrinology, Neurology, Zoology, Animal physiology

## Abstract

Ascidians are the sister group of vertebrates and occupy a critical position in explorations of the evolution of the endocrine and nervous systems of chordates. Here, we describe the complete ventral peptidergic system in adult transgenic *Ciona robusta* (*Ciona intestinalis* Type A) which expresses the *Kaede* reporter gene driven by the prohormone convertase 2 (PC2) gene promoter. Numerous PC2 promoter-driven fluorescent (Kaede-positive) non-neural cells were distributed in the blood sinus located at the anterior end of the pharynx, suggesting the acquisition of a peptidergic circulatory system in *Ciona*. Kaede-positive ciliated columnar cells, rounded cells, and tall ciliated cells were observed in the alimentary organs, including the endostyle, pharynx, esophagus, stomach, and intestine, suggesting that digestive functions are regulated by multiple peptidergic systems. In the heart, Kaede-positive neurons were located in the ring-shaped plexus at both ends of the myocardium. Nerve fiber–like tracts ran along the raphe and appeared to be connected with the plexuses. Such unique structures suggest a role for the peptidergic system in cardiac function. Collectively, the present anatomic analysis revealed the major framework of the ventral peptidergic system of adult *Ciona*, which could facilitate investigations of peptidergic regulation of the pharynx, endostyle, alimentary tissues, and heart.

## Introduction

The phylogenetic position of tunicates as the sister group of vertebrates sheds light on their significance in investigations of the anatomic, molecular, and functional evolution of the chordate nervous and endocrine systems^1–3^. Neuropeptides and peptide hormones are major factors that regulate biological functions in the nervous and endocrine systems. Over the past two decades, a wide variety of neuropeptides and their genes encoding homologs of vertebrate neuropeptides and peptide hormones have been identified in the neural complex of the ascidian *Ciona robusta* (synonym for *Ciona intestinalis* Type A^4^), including cionin, gonadotropin-releasing hormones (GnRHs), tachykinin (Ci-TK), calcitonin, vasopressin (Ci-VP), and insulin-like peptide^5–12^. Furthermore, peptidomic analyses identified more than 30 neuropeptides and peptide hormones, including various *Ciona*-specific peptides as well as homologous peptides from the neural complex of *Ciona*^1,3,7,10,11,13–15^. Some of these neuropeptides and their receptors are expressed in both the central nervous system and peripheral organs, including the ovary, alimentary tissues, endostyle, and heart, suggesting that a variety of peptides participate in the regulation of peripheral organs^7,16^. However, the distribution and morphology of peptidergic cells in the peripheral organs remain to be elucidated.

To investigate the peptidergic systems in peripheral organs, we previously used transgenic *Ciona* that expresses the *Kaede* reporter gene driven by the prohormone convertase 2 (PC2) gene promoter. PC2 is a major protease responsible for the endoproteolytic maturation of peptide hormones and neuropeptides. The PC2 gene is evolutionarily conserved and expressed specifically in major peptidergic systems, including the endocrine, neuroendocrine, and nervous systems of vertebrates and *Ciona*^17,18^. Hence, transgenic *Ciona* harboring the *Kaede* reporter gene construct driven by the PC2 promoter can be used to investigate the morphological features of peptidergic systems. Our recent anatomic analysis of transgenic *Ciona* characterized the dorsal peptidergic system in detail, including the innervations of the anterior and posterior primary nerves to the oral and atrial siphons, the full trajectory of the dorsal strand plexus (DSP), and visceral nerves from the cerebral ganglion to the ovary^19^. We further characterized in detail innervations between the cerebral ganglion and peripheral organs, including the gonoduct, cupular organ, rectum, and ovary in the dorsal part of *Ciona*^19^. Moreover, *Ciona* tachykinin homolog (Ci-TK-I) was shown to specifically enhance the growth of vitellogenic follicles^1,20,21^, and *Ciona* neurotensin-like peptide 6 (Ci-NTLP6) was shown to down-regulate Ci-TK-I–induced oocyte growth^13^. Collectively, data regarding the innervation of peptidergic nerves from the cerebral ganglion to the ovary reinforced the idea that some neuropeptides produced in the neural complex are transported through the nerve fibers and function in the ovary.

In contrast to the dorsal peptidergic system, the ventral peptidergic system remains to be investigated. The endostyle, pharynx, heart, esophagus, stomach, and intestine are located in the ventral region, suggesting that the ventral peptidergic system is essential for the regulation of visceral functions in *Ciona*. In this study, we characterized the peptidergic system in the ventral region using PC2 promoter-driven Kaede-transgenic *Ciona*. Considered in light of our previous data regarding the dorsal nervous system of adult ascidians^19^, those of the present study will contribute significantly to future explorations of the entire peptidergic system of *Ciona* and the evolution of chordate peptidergic systems.

## Materials and Methods

### Transgenic lines

*Ciona robusta* (synonym for *Ciona intestinalis* type A^4^) was used in this study. *Ciona robusta* is referred to as *Ciona* in this manuscript in accordance with the description in the database of transgenic lines of the National BioResource Project of Japan (http://marinebio.nbrp.jp/). The *PC2* > *Kaede* line of *Ciona*, Tg[MiCiPC2K]2, was created by *Minos*-mediated transgenesis^22,23^ and provided by the National BioResource Project. These lines were cultured using the inland culture system as described previously^24^. Adult transgenic animals were used in this study. We observed the general Kaede expression pattern of at least three individual animals and confirmed that all transgenic *Ciona* exhibited essentially identical Kaede expression. We also confirmed identical Kaede expression pattern in another *PC2* > *Kaede* line, Tg[MiCiPC2K]3 for the validation of intrinsic PC2 promoter activity (Fig. S1).

### Tissue preparation

Animals were anesthetized using L-menthol based on a previously reported method^19,25^. In brief, 0.56% (weight per volume) L-menthol in ethanol was prepared and used as a stock solution. The stock solution was further diluted with 1% (volume per volume) artificial seawater before use. Animals were soaked in the diluted L-menthol solution for 10 min. Part of the tunic, body-wall muscle, and pharynx were excised under a fluorescence stereomicroscope (Leica M205 FA; Leica Microsystems, Wetzlar, Germany), and the whole animals were fixed in 4% paraformaldehyde in PBS at 4 °C overnight. The fixed specimens were soaked in 2 mg/ml glycine in PBS to quench the paraformaldehyde. The specimens were then washed three times with PBS and used for morphological analyses. Anatomic analyses were performed under the stereomicroscope (Leica M205 FA; Leica Microsystems) using precision tweezers (Outils Rubis SA, Stabio, Switzerland) and microscissors (Inami & Co., Ltd., Tokyo, Japan).

### Whole-mount observation of tissues

After removal of the tunic, fixed tissues were placed in a glass dish with a thick silicone resin sheet or in a slide glass chamber (AGC TECHNO GLASS, Tokyo, Japan) filled with PBS and secured with needles as appropriate. A fluorescence stereomicroscope (Leica M205 FA; Leica Microsystems) and image acquisition software (Leica AF6000E; Leica Microsystems) were used for low-magnification observations. A confocal microscope with a 40× or 100× objective (FLUOVIEW FV1000; Olympus, Tokyo, Japan) was used for high-magnification observations and three-dimensional image acquisition. To construct three-dimensional images using the confocal microscope, the z-stack function was used, and 12–36 cross-section images of fixed tissues were collected (from top to bottom) per sample. The focus interval was 1 μm for each section image. The section images were merged using FV10-ASW software, version 04.02.03.06 (Olympus), to construct three-dimensional images. To construct deep-focus images using the stereomicroscope, multiple images at various focuses were merged using Adobe Photoshop Elements 13 (Adobe Systems, San Jose, CA, USA). Multiple images were integrated using Adobe Photoshop Elements 13 to construct composites of the low-magnification images. The images were processed using Adobe Photoshop Elements 13 (Adobe Systems) or FV10-ASW software, version 04.02.03.06 (Olympus), to harmonize or optimize brightness and contrast in the composite images or each individual image.

### Freeze sectioning

Fixed tissues were soaked in refrigerated sucrose solution (30% sucrose in PBS) until they sank. The tissues were then embedded in Super Cryoembedding Medium-L1 (Leica Microsystems Japan, Tokyo, Japan) and sectioned at 14- to 16-μm thickness using a CryoStar NX70 cryostat (Thermo Fisher Scientific Inc., Waltham, MA, USA) at −20 °C. The sections were placed onto FRONTIER-coated slides (FRC-04; Matsunami Glass Ind., Ltd., Osaka, Japan) and then mounted using Fluoromount (Diagnostic BioSystems, Pleasanton, CA, USA). A confocal microscope with 20× or 40× objective (FLUOVIEW FV1000; Olympus) was used for observation.

### Hematoxylin-eosin staining

Sections were stained with Mayer’s hemalum solution and 0.5% Eosin Yellowish solution. Mayer’s hemalum solution was diluted 40-fold with distilled water to avoid overstaining. The staining was performed based on the standard protocol. In brief, sections were washed with distilled water for 5 min and immersed in Mayer’s hemalum solution for 10 min. The sections were then washed with flowing water for 5 min and further immersed in Eosin solution for 2 min. After rinsing with distilled water, the sections were dehydrated using an alcohol series composed of 70%, 80%, 95%, and 100% ethanol and xylene. The sections were finally sealed with a cover glass (Matsunami Glass Ind., Ltd.) and MOUNT-QUICK (Daido Sangyo Co., Ltd., Tokyo, Japan) and observed under a light microscope with a 20× objective (Axio Imager 2; Carl Zeiss, Oberkochen, Germany) in phase-contrast mode.

## Results and Discussion

### Overview of the distribution of peptidergic cells in the ventral part of transgenic *Ciona*

**Figure 1:**
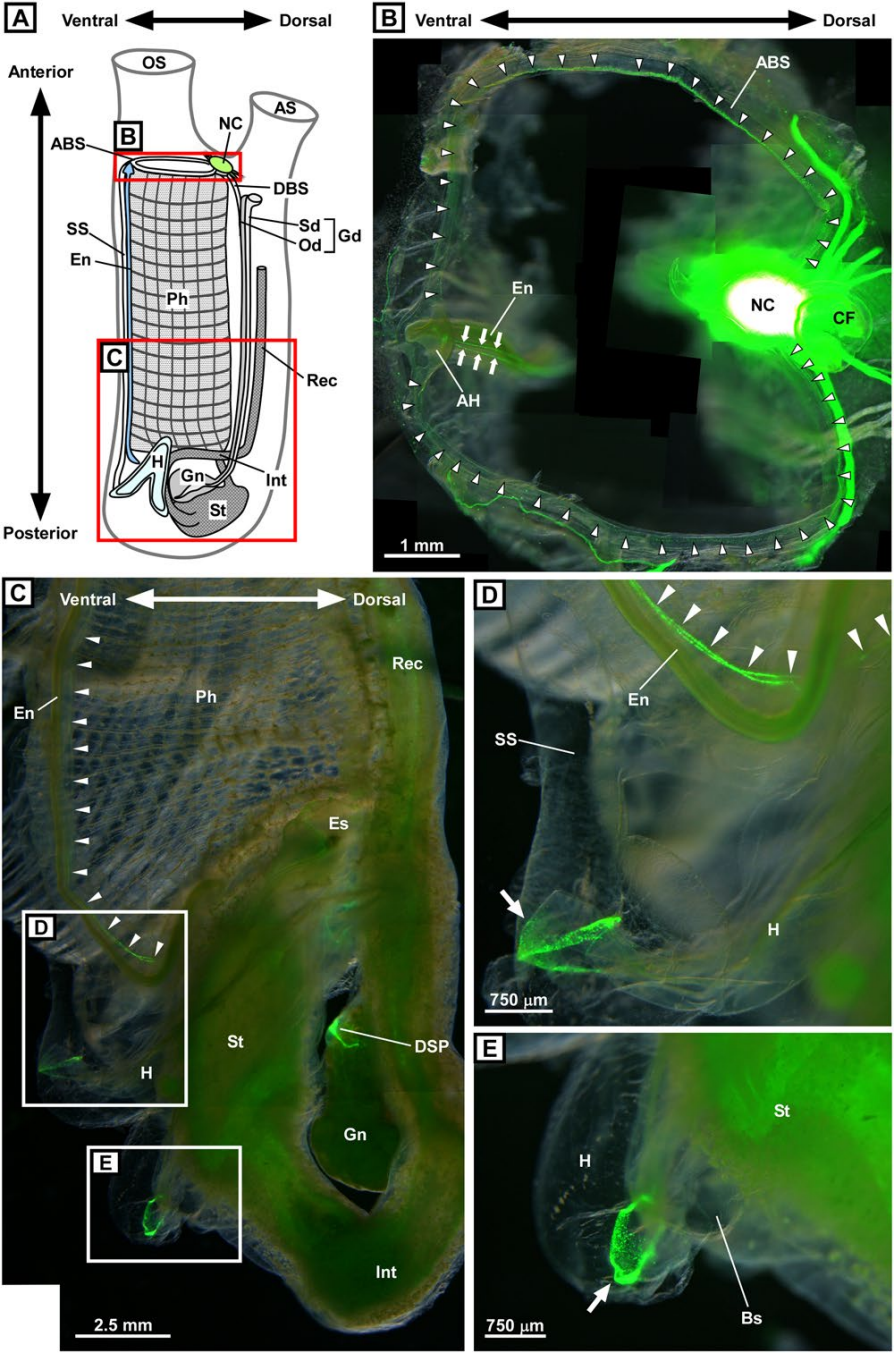
Overview of the distribution of PC2 promoter-driven Kaede-positive cells in transgenic *Ciona*. (**A**) Schematic illustration of an adult *Ciona*. The key anatomic parts of an adult *Ciona* are indicated by different fill patterns. (**B**) Kaede-positive cells in the framed region shown in (**A**). The anterior end of the pharynx viewed from above is shown. Arrowheads indicate Kaede-positive cells distributed along the anterior pharyngeal blood sinus located at the anterior end of the pharynx. Arrows indicate Kaede-positive cells in the endostyle. The oral siphon was removed to make the blood sinus more visible. Thirty-one images were merged to construct a composite image. The neural complex has a very strong Kaede-positive signal that was described in the previous study^18^. Note that the ciliated funnel is not Kaede-positive, but Kaede signals from the neural complex and nerve fibers are shown through the ciliated funnel. (**C**) Kaede-positive cells in the posterior half of the transgenic *Ciona* framed in (**A**). Peripheral organs, including the endostyle, pharynx, heart, and stomach are shown. Arrowheads indicate Kaede-positive cells in the endostyle. Kaede-positive signals were also observed in the heart, stomach, and dorsal strand plexus. Kaede-positive signals in the pharynx were relatively weak and not visible in this image. The tunic, body-wall muscle, and part of the pharynx were removed to make the peripheral organs more visible. Seven images were merged to construct a composite image. (**D**) Magnified image of the framed region in (**C**). The junction between the heart and blood sinus adjacent to the endostyle is shown. The left outlet of the heart is surrounded by Kaede-positive cells (arrow). Kaede-positive cells in the endostyle are indicated by arrowheads. Two images were merged to construct a composite image. (**E**) Magnified image of the framed region in (**C**). The junction between the heart and blood sinus connecting to the stomach is shown. The right outlet of the heart is surrounded by Kaede-positive cells. These images were obtained under a fluorescence stereomicroscope as superimposed images. ABS, anterior pharyngeal blood sinus, AH, anterior hood; AS, atrial siphon; BS, blood sinus; CF, ciliated funnel; DBS, dorsal blood sinus; DSP, dorsal strand plexus; En, endostyle; Es, esophagus; Gd, gonoduct; Gn, gonad; H, heart; Int, intestine; NC, neural complex; Od; oviduct, OS, oral siphon; Ph, pharynx; Rec, rectum; Sd, spermiduct; SS, subendostylar sinus; St, stomach.

**Figure 2:**
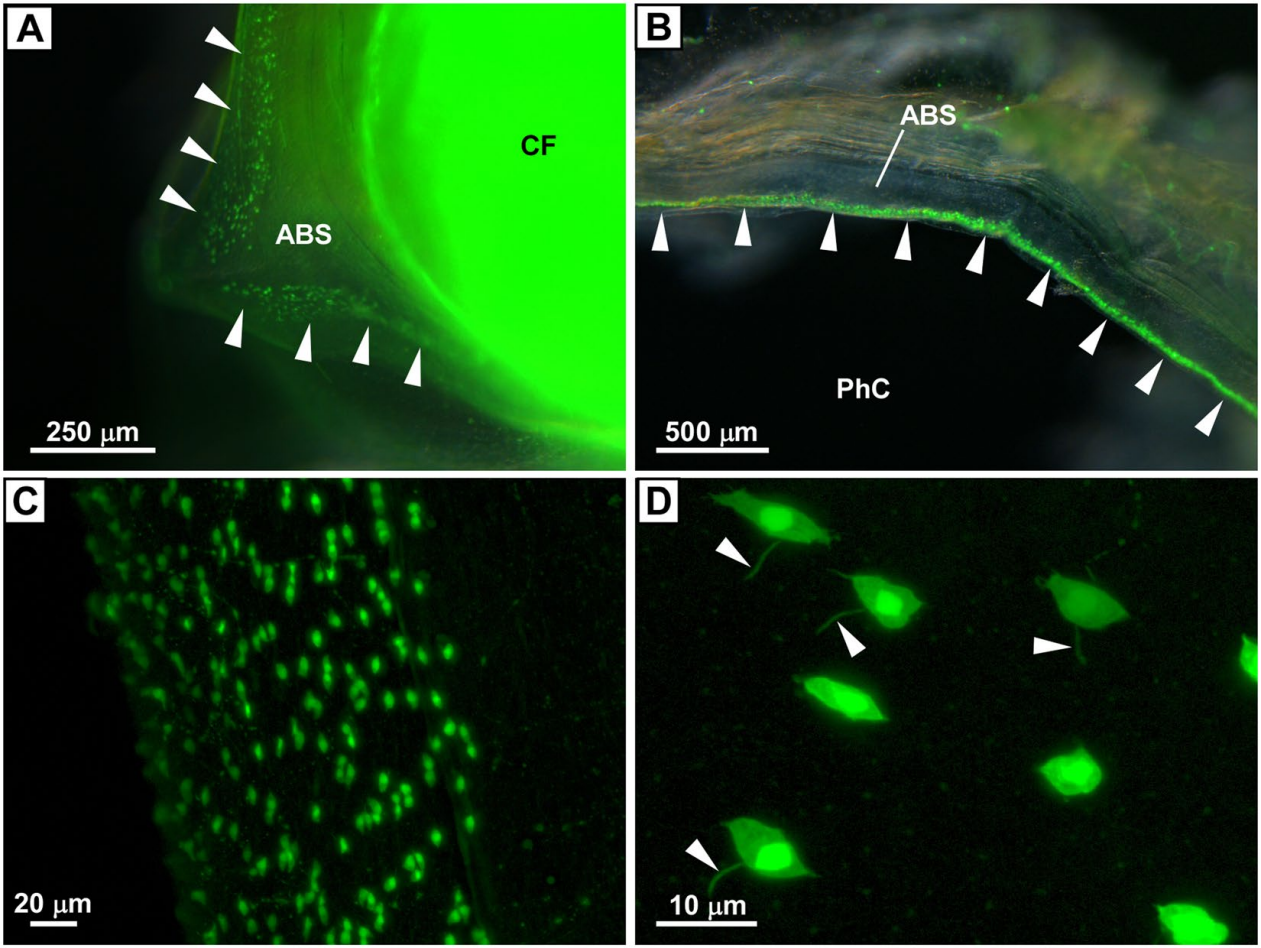
Distribution of PC2 promoter-driven Kaede-positive cells in the blood sinus. (**A**) Kaede-positive cells in the anterior pharyngeal blood sinus adjacent to the ciliated funnel of the neural complex. The blood sinus and ciliated funnel of the neural complex viewed from above are shown. Arrowheads indicate Kaede-positive cells. Note that the ciliated funnel is not Kaede-positive, but Kaede signals from the neural complex and nerve fibers are shown through the ciliated funnel. (**B**) Kaede-positive cells in the lateral part of the anterior pharyngeal blood sinus. Kaede-positive cells were distributed in the area facing the pharyngeal cavity (arrowheads). (**C**) Magnified image of the anterior pharyngeal blood sinus. Numerous spindle-shaped Kaede-positive cells are shown. (**D**) Magnified image of Kaede-positive cells. Spindle-shaped Kaede-positive cells with primary cilia-like structures (arrowheads) are shown. The images in (**A**,**B**) were obtained under a fluorescence stereomicroscope as a superimposed image. The images in (**C**,**D**) were obtained as z-stack images under a confocal laser scanning microscope. ABS, anterior pharyngeal blood sinus; CF, ciliated funnel; PhC, pharyngeal cavity.

**Figure 3:**
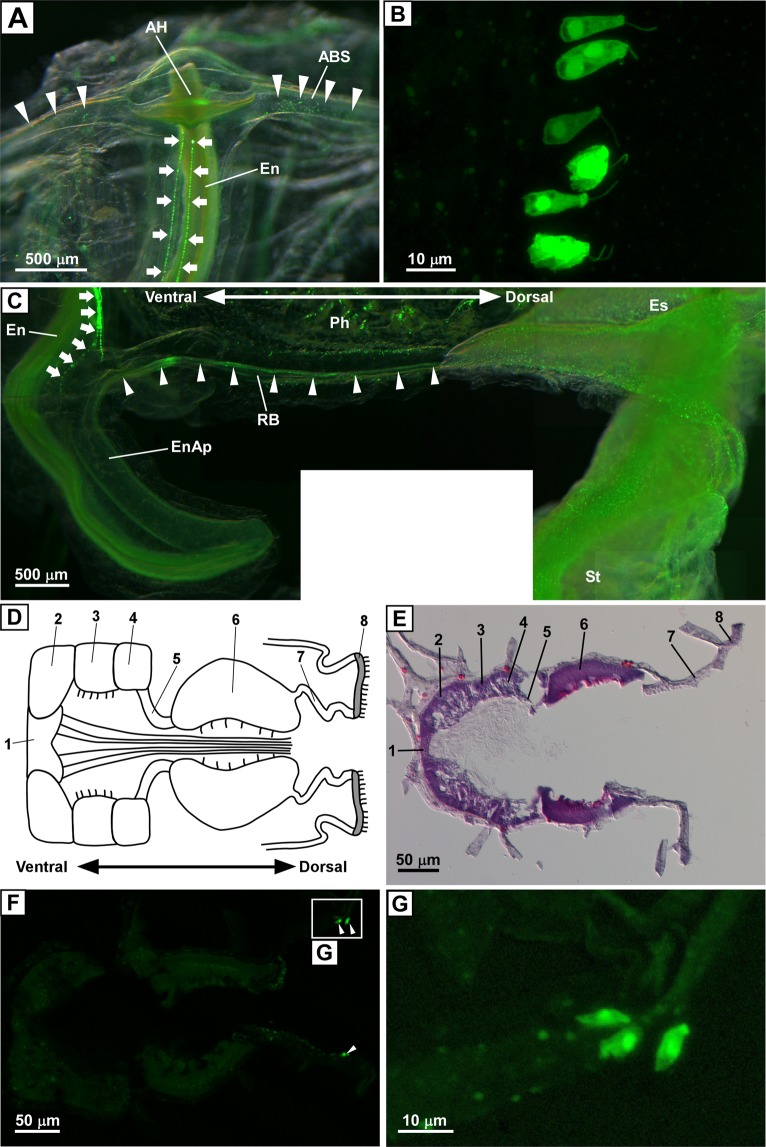
Distribution of PC2 promoter-driven Kaede-positive cells in the endostyle. (**A**) Kaede-positive cells at the anterior end of the endostyle. Front view of the anterior end of the endostyle is shown. Arrows indicate Kaede-positive cells aligning in two rows in the endostyle. Arrowheads indicate Kaede-positive cells in the anterior pharyngeal blood sinus. The oral siphon was removed to make the endostyle more visible. Four images were merged to construct a deep-focus image. (**B**) Magnified image of the Kaede-positive cells in the endostyle. Kaede-positive ciliated cells in one row are shown. (**C**) Kaede-positive cells in the posterior end of the endostyle and pharynx. The posterior end of the endostyle and pharynx, and the endostylar appendix that extends out of the pharynx, are shown. Kaede-positive cells in the endostyle were observed continuously near the posterior end of the pharynx, and a small number of the cells was observed in the endostylar appendix (arrows). Arrowheads indicate Kaede signals along the blood sinus, which is also known as the retropharyngeal band. Fourteen images were merged to construct a composite image. (**D**) Schematic illustration of the horizontal section of the endostyle. Zone names are indicated by numbers. Short and long cilia are indicated by solid lines. (**E**) Hematoxylin-eosin staining of the horizontal section of the endostyle. Zone names are indicated by numbers. (**F**) Fluorescent image of the horizontal section of the endostyle. Kaede-positive cells in zone 7 are indicated by arrowheads. (**G**) Magnified image of the framed region in (**F**). Kaede-positive cells are localized in the dorsal terminal of zone 7, proximal to zone 8. The images in (**A**,**C**) were obtained under a fluorescence stereomicroscope as a superimposed image. The images in (**B**,**F**,**G**) were obtained under a confocal laser scanning microscope, and the image in (**B**) was obtained as a z-stack image. The image in (**E**) was obtained under a light microscope. ABS, anterior pharyngeal blood sinus; AH, anterior hood; En, endostyle; EnAp, endostylar appendix; Es, esophagus; Ph, pharynx; RB, retropharyngeal band; St, stomach.

**Figure 4:**
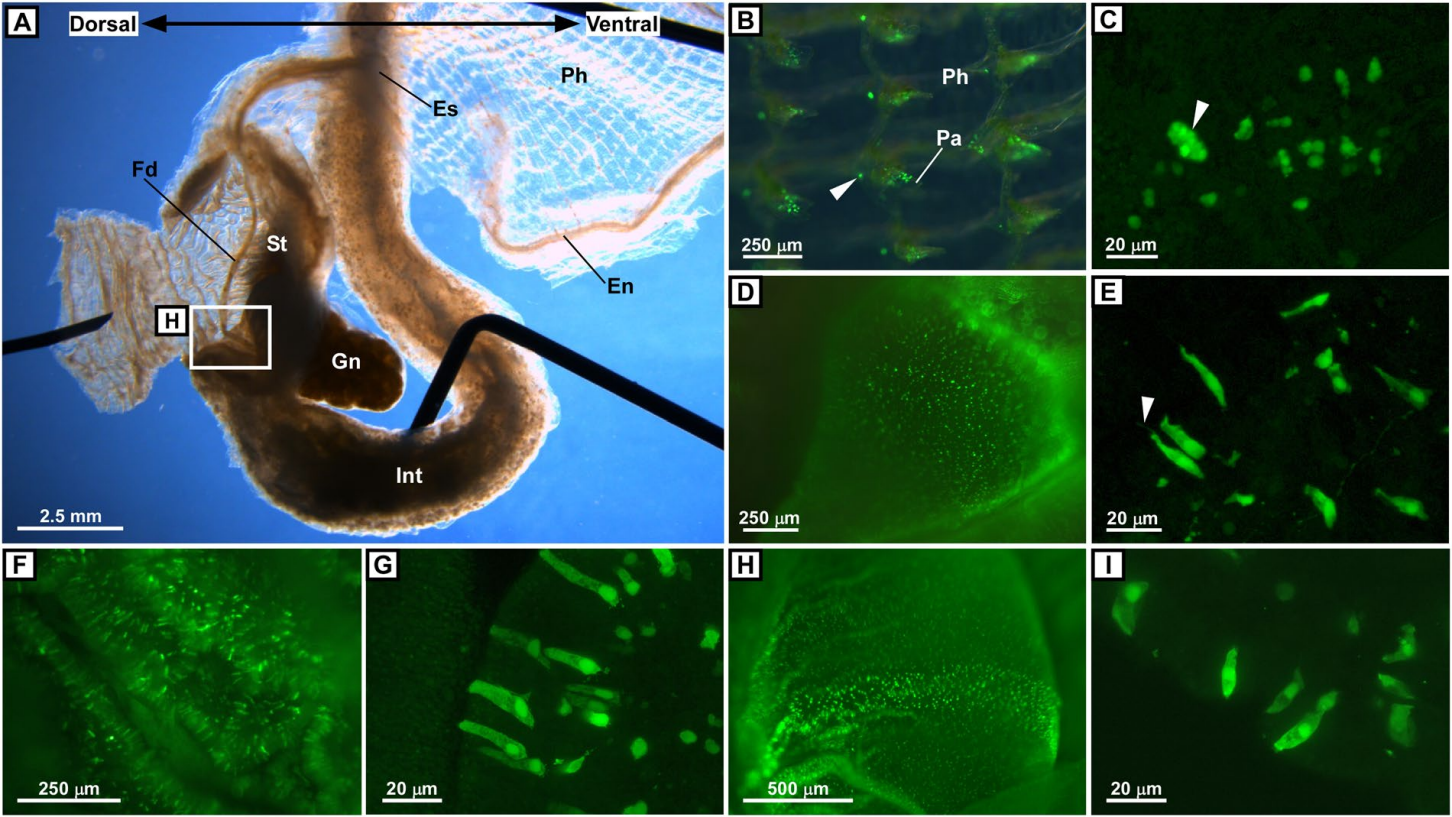
Distribution of PC2 promoter-driven Kaede-positive cells in the alimentary system. (**A**) Overview of the alimentary organs. The pharynx, esophagus, stomach, and intestine are shown in a bright-field image. Part of the stomach is dissected to show the inside wall. Note that the direction of the dorsal-ventral axis is opposite to other figures. Needles used to fix the tissues are shown in black. A food cord is present in the stomach. (**B**) Kaede-positive cells in the pharynx. Kaede-positive cells in each papilla of the pharynx are shown. A typical cell cluster at the bottom of the papilla is indicated by an arrowhead. (**C**) Magnified image of Kaede-positive cells in a papilla. Small, rounded Kaede-positive cells dispersing in the papilla and a cell cluster at the bottom of the papilla (arrowhead) are shown. (**D**) Kaede-positive cells in the esophagus. Numerous Kaede-positive cells uniformly distributed in the uppermost part of the esophagus are shown. (**E**) Magnified image of the Kaede-positive cells in the esophagus. Tall, ciliated Kaede-positive cells constructing the esophagus epithelium are shown. The arrowhead indicates a cilium. (**F**) Kaede-positive cells in the stomach. Numerous Kaede-positive cells distributing in the folds of the stomach are shown. Three images were merged to construct a deep-focus image. (**G**) Magnified image of the Kaede-positive cells in the stomach. Tall Kaede-positive cells constructing the stomach epithelium are shown. (**H**) Kaede-positive cells in the lower part of the stomach. Kaede-positive cells uniformly distributed in the lower part of the stomach are shown. Four images were merged to construct a deep-focus image. (**I**) Magnified image of the Kaede-positive cells in the intestine. Tall Kaede-positive cells constructing the intestine epithelium are shown. The images in (**A**,**B**,**D**,**F**,**H**) were obtained under a fluorescence stereomicroscope. The images in (**C**,**E**,**G**,**I**) were obtained as z-stack images under a confocal laser scanning microscope. En, endostyle; Es, esophagus; Fd, food cord; Gn, gonad; Int, intestine; Pa, papilla; Ph, pharynx; St, Stomach.

**Figure 5:**
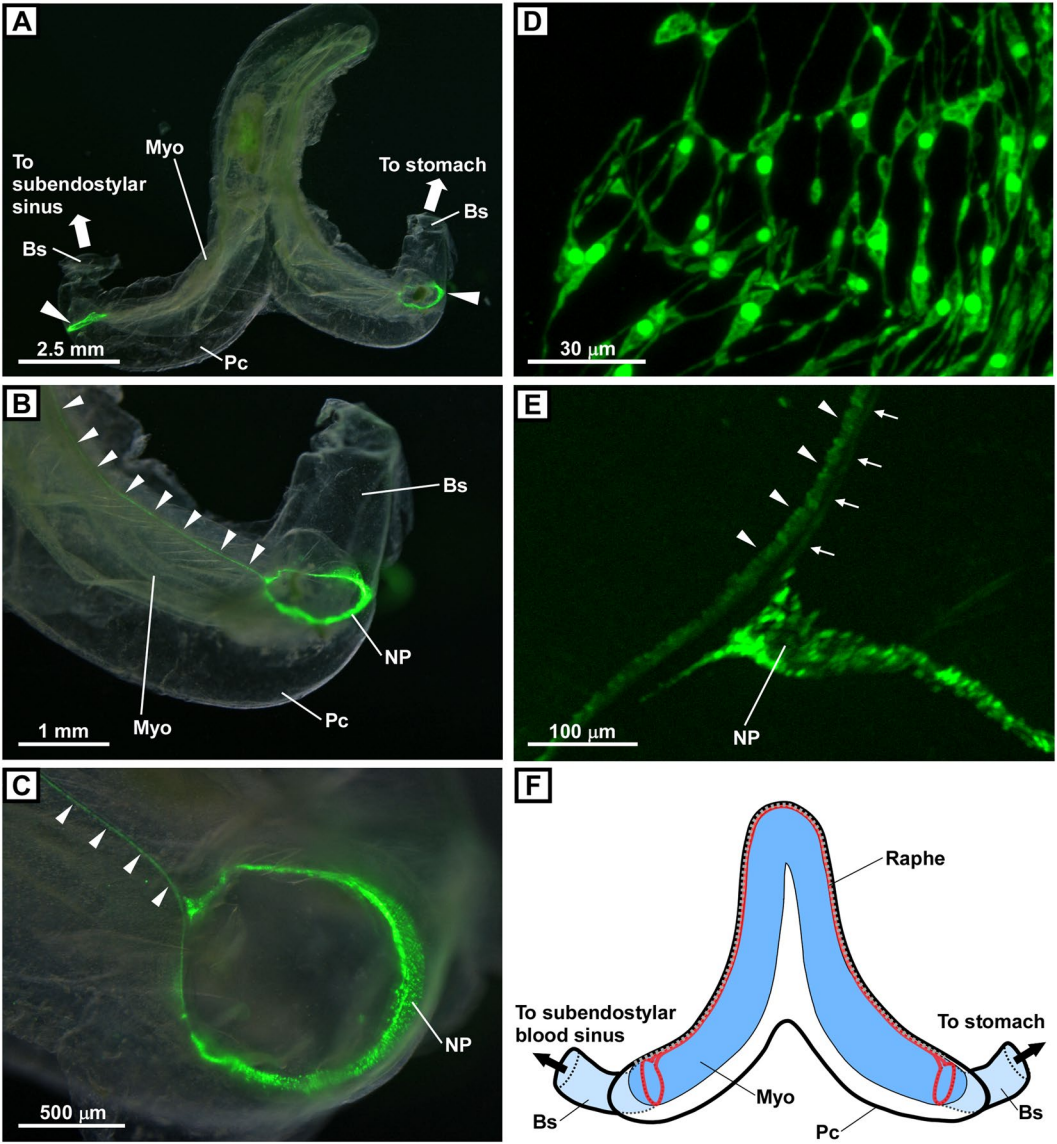
PC2 promoter-driven Kaede-positive cells in the heart. (**A**) Overview of the heart. The myocardium surrounded by the pericardium and blood sinuses connecting to both terminals of the heart are shown. Kaede-positive cells forming a ring-shaped structure at both terminals of the heart are shown (arrowheads). (**B**) Magnified image of the right terminal of the heart. The ring-shaped structure of the Kaede-positive cells in the terminal of the myocardium is shown. Kaede signals along the raphe are also shown (arrowheads). (**C**) Magnified image of the right terminal of the heart. The ring of the Kaede-positive cells appeared not to be closed, and a major Kaede-positive tract emerged from one end of the ring and runs along the raphe (arrowheads). (**D**) Magnified image of the Kaede-positive cells in the ring-shaped nerve plexus in the heart. Numerous Kaede-positive neurons forming networks are shown. (**E**) Magnified image of the Kaede-positive tract emerging from the nerve plexus. Major and minor Kaede-positive tracts are indicated by arrowheads and arrows, respectively. (**F**) Schematic illustration of the heart. The myocardium is shown in blue. Blood sinuses are shown in light blue. The raphe, which connects the pericardium and myocardium, is indicated by a gray dotted line. The ring of Kaede-positive cells and tracts are indicated by a red line. The images in (**A**–**C**) were obtained under a fluorescence stereomicroscope as a superimposed image. The images in (**D**,**E**) were obtained under a confocal laser scanning microscope, and the image in (**D**) was obtained as a z-stack image. Bs, blood sinus; Myo, myocardium; NP, nerve plexus; Pc, pericardium.

The present study examined the distribution of PC2 promoter-driven Kaede-positive cells in the peripheral organs, including the blood sinus, endostyle, pharynx, heart, stomach, and intestine, located in the ventral part of transgenic adult *Ciona*. Figure 1 shows an overview of the distribution of Kaede-positive cells in the ventral area of transgenic adult *Ciona*. The pharynx, one of the largest organs in *Ciona*, collects foods from seawater. The pharynx is also the site of gaseous exchange with seawater, and pharyngeal blood vessels run throughout the pharynx^26^. A large blood sinus, also known as the peripharyngeal band, is located at the anterior end of the pharynx^27^. This blood sinus (called the ‘anterior pharyngeal blood sinus’ in this study) connects to the subendostylar sinus on the ventral side as well as to the dorsal blood sinus on the dorsal side of the pharynx (Fig. 1A). Kaede-positive cells were specifically distributed in the anterior pharyngeal blood sinus (Fig. 1A,B). On the ventral side of the pharynx, the endostyle extends antero-posteriorly. The endostyle secretes mucus proteins for internal filter feeding^26^. Kaede-positive cells were aligned in two rows in the endostyle (Fig. 1B) and located continuously from the anterior end almost to the posterior end of the endostyle (Fig. 1C,D). At the posterior end of the pharynx, the heart lies adjacent to the stomach and posterior end of the endostyle (Fig. 1A,C). The left terminal of the heart connects to the subendostylar sinus, which is also called the hypobranchial vessel^26,28^, and the right terminal of the heart connects to the stomach via a short blood sinus (Fig. 1D,E). Kaede-positive cells were specifically observed in the junctions between the left terminal of the heart and the subendostylar vessel (Fig. 1C) and between the right terminal of the heart and the stomach (Fig. 1E). In the pharynx, esophagus, stomach, and intestine, relatively weak Kaede signals were observed in a low-magnification view (Fig. 1C). The precise distribution of Kaede-positive cells in the ventral organs is shown in the following figures (Figs. 2–5). Kaede-positive cells were also distributed in the DSP (Fig. 1C), as reported in our previous study^19^.

### Distribution of peptdergic cells in the blood sinus

The distribution of Kaede-positive cells in the anterior pharyngeal blood sinus began near the ciliated funnel of the neural complex and continued to near the ventral end of the blood sinus, where the sinus integrates with the endostyle (Figs. 1B and 2A). Both low- and high-magnification images showed that spindle-shaped Kaede-positive cells were distributed on the wall of the blood sinus facing the pharyngeal cavity (Fig. 2B,C). These cells did not appear to project axons, suggesting that they are non-neural endocrine cells (Fig. 2C,D). Interestingly, each cell extended a cilium less than 10 µm from the middle of the cell, suggesting that these cilia are primary cilia (Fig. 2D). Primary cilia-like structures were also reported in the endodermal strand of *Ciona* tadpoles^29^. Although primary cilia are considered rare or non-existent in non-neuronal cells in invertebrates^30^, the results of the present study suggest that *Ciona* possesses primary cilia in non-neuronal cells. Because the ciliated Kaede-positive cells face the pharyngeal cavity, through which food and seawater pass, the cilia may be involved in the transmission of outer environment substances and/or biological signaling as well as the regulation of various cellular and physiological processes, as in vertebrates^31^. No Kaede-positive cells were observed in the blood sinuses of other areas, suggesting that the anterior pharyngeal blood sinus plays a specific endocrine role in the circulatory system. To our knowledge, this is the first report showing the presence of Kaede-positive cells in the blood sinus of an ascidian or Kaede-positive cells with cilia-like structures. In vertebrates, endothelin, a potent vasocontstrictor peptide^32,33^, and adrenomedullin, a potent hypotensive peptide, are produced in and secreted from endothelial cells of blood vessels^34^. Moreover, perivascular cells are responsible for synthesis and release of alarin, a peptide yielded from a splice variant of the galanin-like peptide (GALP) gene in mammals^35^. In combination, the present study indicates that *Ciona* shares similar peptidergic regulations in some peptidergic circulatory system with vertebrates, and suggests that such regulatory systems might have been established in ancestral chordates. Identification of endogenous peptides in the Kaede-positive cells of the *Ciona* blood sinus and the elucidation of their physiological functions will provide further insights into the evolutionary process of peptidergic circulatory systems in chordates.

### Distribution of peptidergic cells in the endostyle

On the ventral side of the pharynx, the endostyle extends antero-posteriorly. The endostyle is integrated with the anterior pharyngeal blood sinus at its anterior end, which is called the anterior hood^27^. Kaede-positive cells arranged in two rows were observed starting near the anterior hood and continuing posteriorly throughout the endostyle (Fig. 3A). A magnified image clearly showed that the ciliated columnar cells were arranged in a line (Fig. 3B). The lack of axons in these cells suggests that they are non-neural endocrine cells. Kaede-positive-ergic cells were continuously observed in the endostyle proximal to the posterior end of the pharynx, and the distribution of these cells terminated at the posterior end of the endostyle (Fig. 3C), which is called the endostylar appendix^27^. The endostylar appendix is a unique structure occurring only in *C. intestinalis*^27^. Although the functions of the endostylar appendix are unclear, the almost complete absence of fluorescence suggested that this area is not subjected to peptidergic regulation. Weak Kaede-positive fluorescent signals were also observed in the blood sinus located at the bottom of the pharynx, which is also known as the retropharyngeal band, and these fluorescent signals continued to the esophagus (Fig. 3C). Our observations suggest that this blood sinus functions in the peptidergic system; however, whether small Kaede-positive cells or nerve fibers are distributed in this region could not be determined due to very low level of fluorescence.

To determine the precise localization of Kaede-positive cells in the endostyle, we further examined the histological structure of the endostyle using tissue sections. Hematoxylin-eosin staining clearly showed the eight zones of the endostyle in a horizontal section (Fig. 3D,E), as previously reported^26^. Kaede-positive cells were localized in zone 7, proximal to zone 8 (Fig. 3F,G). Interestingly, this narrow area is coincident with the area that expresses mRNA encoding Ci-TK, a neuropeptide identified in the neural complex of *Ciona*^7^. No Kaede-positive cells were observed in other zones, suggesting that zone 7 specifically produces and releases Ci-TK. The eight zones of the endostyle are thought to have different functions. Generally, zones 1, 3, 5, and 8 of the endostyle are thought to spread and laterally transport secretions from other zones^26^. Zones 2, 4, 6, and 7 are known to be secretory zones that produce muco-protein, and zone 7 is specifically involved in the addition of iodinated compounds^26,36^. Indeed, mRNA of thyroid peroxidase, an iodination enzyme, is expressed throughout zone 7^37^. Collectively, these results indicate that peptidergic cells are localized in a restricted area of zone 7 and that some peptidergic systems, including Ci-TK, may participate in the iodination of muco-proteins.

Urochordates, cephalochordates, and larval lampreys have the endostyle, which possesses iodine-metabolic functions and expresses the thyroid-related transcription factors^38–41^. In addition, the endostyle is transformed into the follicular thyroid during metamorphosis in lampreys^42^. These findings suggest that the endostyle is an ancestral or precursor organ of the vertebrate thyroid gland^41^. Furthermore, the expression of PC2 and some peptides including calcitonin (CT), somatostatin, and ghrelin was observed in the thyroid gland of mammals and the ultimobranchial gland of non-mammalian vertebrates (the counterpart for mammalian thyroid glands)^43,44^. A main function of peptidergic system in the thyroid gland and ultimobranchial gland is calcium homeostasis that is controlled by CT^45^. Interestingly, calcitonin mRNA was expressed in the *Ciona* endostyle^12^. The *Ciona* calcitonin (Ci-CT) reduced the osteoclastic activity on goldfish scales that is a typical action of vertebrate calcitonin^12^. These findings suggest that the peptidergic system in the endostyle/ultimobranchial gland/thyroid gland might have been established in ancestral chordates. The mineral homeostasis functions might have been conserved in the chordate peptidergic systems in the endostyle/ultimobranchial gland/thyroid gland.

### Distribution of peptidergic cells in the alimentary system

The distribution of Kaede-positive cells was also investigated in the alimentary system, including the pharynx, esophagus, stomach, and intestine (Fig. 4). In the pharynx, small, rounded Kaede-positive cells were scattered in each papilla, and a cluster of the rounded cells was located at the base of each papilla (Fig. 4B,C). Papillae are thought to assist in passing the mucus sheet produced in the endostyle across the pharyngeal wall from the ventral to the dorsal side, and the mucus sheet containing food is folded into a mucus cord by the dorsal lamina^26^. Given that genes encoding Ci-CT, Ci-LF-8 receptor, Ci-YFV-1 receptor, and Ci-YFV-3 receptor, are expressed in the pharynx^12,46^, multiple peptides may be involved in regulating the food transport function of the papillae.

There is no consensus regarding the types of *Ciona* esophagus epithelial cells. An earlier study reported that the esophagus epithelium consists of two types of cells, tall ciliated cells and mucus-secreting cells^47^. A subsequent study reported that the esophagus epithelium is comprised of only ciliary-mucus cells^48^. The results of the present study indicate that many—but not all—of the epithelial cells are tall ciliated Kaede-positive cells (Fig. 4D,E) and that the esophagus epithelium consists of at least two types of cells, tall ciliated Kaede-positive cells and mucus-secreting cells. The peptidergic system in the esophagus may function in food transportation or digestion, although the expression of peptides and their receptors in the esophagus has not been investigated.

Numerous Kaede-positive cells were distributed in the folds of the stomach wall (Fig. 4F). As observed in the esophagus, tall PC2-ergic cells having a basal nucleus were aligned in the folds (Fig. 4G). Tall Kaede-positive cells were uniformly distributed in the wall of the intestine (Figs. 4H,I and S2). The epithelium of the stomach consists of two types of cells: absorption cells and secretory cells^47^. The tall Kaede-positive cells in the stomach or intestine identified in this study appear to be equivalent to the secretory cells or mucus cells described in the earlier study, based on morphological similarity^47^. A recent study reported the expression of secretory digestive enzyme genes and absorption-related protein genes in the stomach and intestine^37^. In addition, innate immune system–related genes, including genes encoding Toll-like receptor and intelectin, are expressed in the stomach and intestine^3,49,50^. These findings indicate that the stomach and intestine of *Ciona* function in both digestion and immunity.

Regarding the expression of neuropeptides and their receptors, homologous peptide and receptor genes of vertebrates including Ci-TK^7^, cionin (a cholecystokinin/gastrin homolog peptide)^16^, and Ci-GALP^13,46^, are known to be expressed in the alimentary system. In particular, *in situ* hybridization showed that the Ci-TK mRNA is expressed in the intestinal epithelial layers^7^. Furthermore, genes encoding the receptors for *Ciona*-specific peptides, Ci-LF-8 receptor and Ci-YFV-3 receptor, are also expressed in the stomach and intestine^46^. Although the localization of these peptides and receptors has yet to be rigorously investigated, the broad distribution of Kaede-positive cells in the stomach and intestine suggests that certain neuropeptides, including Ci-TK, cionin, Ci-GALP, Ci-LF-8, and Ci-YFV-3, participate in regulating all functions of the stomach and intestine. Interestingly, these peptide and receptor genes are expressed also in the neural complex^13,46^, suggesting that the peptidergic systems regulate the brain-stomach and brain-gut axis in *Ciona* as well as in vertebrates^51^. Recently, gastrin-releasing peptide (GRP) gene was identified in the two amphioxus species, other invertebrate chordates^52,53^. The amphioxus GRP mRNA was expressed in the alimentary tract as well as the cerebral vesicle^53^. Altogether, these findings suggest that the peptidergic regulation of the brain-stomach and brain-gut axis is a conserved property in chordates.

### Peptidergic system in the heart

The heart of *Ciona* consists of a myocardium comprised of a field of myoepithelial contractile cells and a pericardium comprised of a monolayer of non-contractile epithelial cells^54,55^. The myocardium is enclosed by the pericardium, and they are connected at the raphe, which runs along the dorsal side of the pericardium^28^. The undifferentiated line lies opposite the raphe and runs along the ventral side of the pericardium^28^. Interestingly, the present study showed that Kaede-positive cells form a ring at both terminals of the myocardium (Fig. 5A–C). Furthermore, Kaede signals formed a tract that ran along the raphe (Fig. 5B,C). A magnified image showed that the ring consisted of Kaede-positive cells (Fig. 5D) that appeared to be bipolar neurons forming a nerve plexus. In previous study, both ends of the heart were characterized as a growth zone in which small cells form a ring^56,57^. Although the identity of the cells forming the rings shown in the present and previous studies remains to be determined, the present study showed for the first time the presence of peptidergic neural networks at both terminals of the myocardium. To date, little attention has been paid to the physiological significance of the raphe^26^. However, the results of the present study clearly showed that Kaede signals form a tract along the raphe (Fig. 5B,C), and these tracts were separated into major and minor tracts that were derived from the nerve plexus (Fig. 5E). The tracts appeared to connect the peptidergic rings of both ends of the heart, suggesting that the raphe plays an important role in regulating the heart.

The tunicate heart is controlled by pacemakers located at either end of the heart, and heart beat reversal occurs in a regular manner, although neither the identity of the pacemaker cells nor the underlying mechanism have been elucidated^26^. The cells at each end of the heart are thought to be involved in pacemaker activity, and external stimuli provided by accumulating pressure or nervous input appear to play roles in heart beat reversal^57^. However, the innervation of the tunicate heart remains unclear, and theories explaining heart beat reversal are controversial^26,57^. The results of the present study clearly showed rings of Kaede-positive neurons at either end of the heart, suggesting that the peptidergic system is a promising candidate pacemaker of the heart. In addition, the Kaede-positive tracts running along the raphe support the hypothesis that the rings of Kaede-positive neurons at either end of the heart exchange information via the raphe, and that the dominance of the pacemaker activity between the two ends of the heart is reversed by desensitization of the Kaede-positive neurons or by various stimulants released from the neurons. mRNAs encoding several peptides and receptors, including Ci-YFV/L, Ci-YFV-3 receptor, Ci-GALP receptor, and Ci-LF-8 receptor, are expressed in the heart of *Ciona*^13,57^, suggesting that these peptides play important roles in regulating heart function. Furthermore, the heart and stomach and the stomach and ovary were found to be connected by blood sinuses (Figs. 1 and S3). Therefore, the peptidergic neurons in the *Ciona* heart may also release peptides into the circulatory system to regulate the functions of the stomach, ovary, and other organs.

Interestingly, major nerve plexuses, termed sinoatrial plexus and atrioventricular plexus, surround the junctions of the sinoatrial valve and atrium and those of atrium and ventricle where the pacemaker cells exist in zebrafish^58,59^. The morphological feature and locations of the nerve plexuses of *Ciona* heart is reminiscent of those of zebrafish, suggesting that such cardiac nervous systems may be in part conserved in *Ciona*.

In conclusion, we characterized the peptidergic system in the ventral part of *Ciona*, including the anterior pharyngeal blood sinus, endostyle, pharynx, esophagus, stomach, intestine, and heart. The present study showed that the ventral organs are regulated primarily by various non-neural peptidergic cells, including small endocrine cells with primary cilia-like structures and tall ciliated endocrine cells, in contrast to the dorsal organs such as the rectum and ovary, which are regulated by the peptidergic nervous system of the brain-dorsal strand axis^19^. Moreover, the *Ciona* heart, unlike the aforementioned ventral organs, is likely regulated by cardiac peptidergic neurons. Integration of the results of the present study with those of our previous study in which we characterized the dorsal peptidergic nervous system^19^ will substantially enhance understanding of the complete peptidergic system in *Ciona*.

## Supplementary information

Supplementary information.

## Data Availability

The datasets generated and/or analyzed during the current study are available from the corresponding author upon reasonable request.
