# Supplementary material for: The ventral peptidergic system of the adult ascidian Ciona robusta (Ciona intestinalis Type A) insights from a transgenic animal model
Source: Sci Rep. 2020 Feb 5;10:1892. doi: 10.1038/s41598-020-58884-w (PMC7002689; doi:10.1038/s41598-020-58884-w)
Supplement: Supplementary file 1 — Supplementary information. [file 41598_2020_58884_MOESM1_ESM.pdf]

**Supplementary information**

# The ventral peptidergic system of the adult ascidian *Ciona robusta* (*Ciona intestinalis* Type A) insights from a transgenic animal models

Tomohiro Osugi, Yasunori Sasakura, Honoo Satake

## Supplementary Figure Legends

**Figure S1.** Distribution of PC2 promoter-driven Kaede-positive cells in another PC2 transgenic line, Tg[MiCiPC2K]3. **(A)** Kaede-positive cells distributed along the anterior pharyngeal blood sinus (arrowheads). The Kaede-positive cells are also shown in the endostyle (arrows). **(B)** Kaede-positive cells in the endostyle. **(C)** Representative Kaede-positive cells in the papilla of the pharynx (arrowheads). A representative cluster of the Kaede-positive cells is indicated by an arrow. **(D)** Kaede-positive cells in the esophagus. **(E)** Kaede-positive cells in the stomach. **(F)** Kaede-positive cells in the intestine. **(G)** Overview of the heart in a composite image. **(H)** Overview of the heart in a dark field image. Kaede signals in both ends of the heart and raphe are shown in green. **(I)** Kaede-positive nerve plexus at the end of the heart. ABS, anterior pharyngeal blood sinus; AH, anterior hood, CF, ciliated funnel; En, endostyle; NC, neural complex.

**Figure S2.** Overview of the alimentary canal of *Ciona*, Tg[MiCiPC2K]2 line. (**A**) Bright-field image of the alimentary canal. The stomach and intestine were cut and opened to show the inner walls. (**B**) Dark-field image of the alimentary canal. Kaede-positive cells are shown as green dots. Gn, gonad; Int, intestine, St, stomach.

**Figure S3.** Blood sinus connecting the stomach and ovary. Bright-field image of the stomach, ovary, and intestine. The stomach and ovary are connected via a blood sinus (arrowheads). St, stomach; Ov, ovary; Int, intestine.

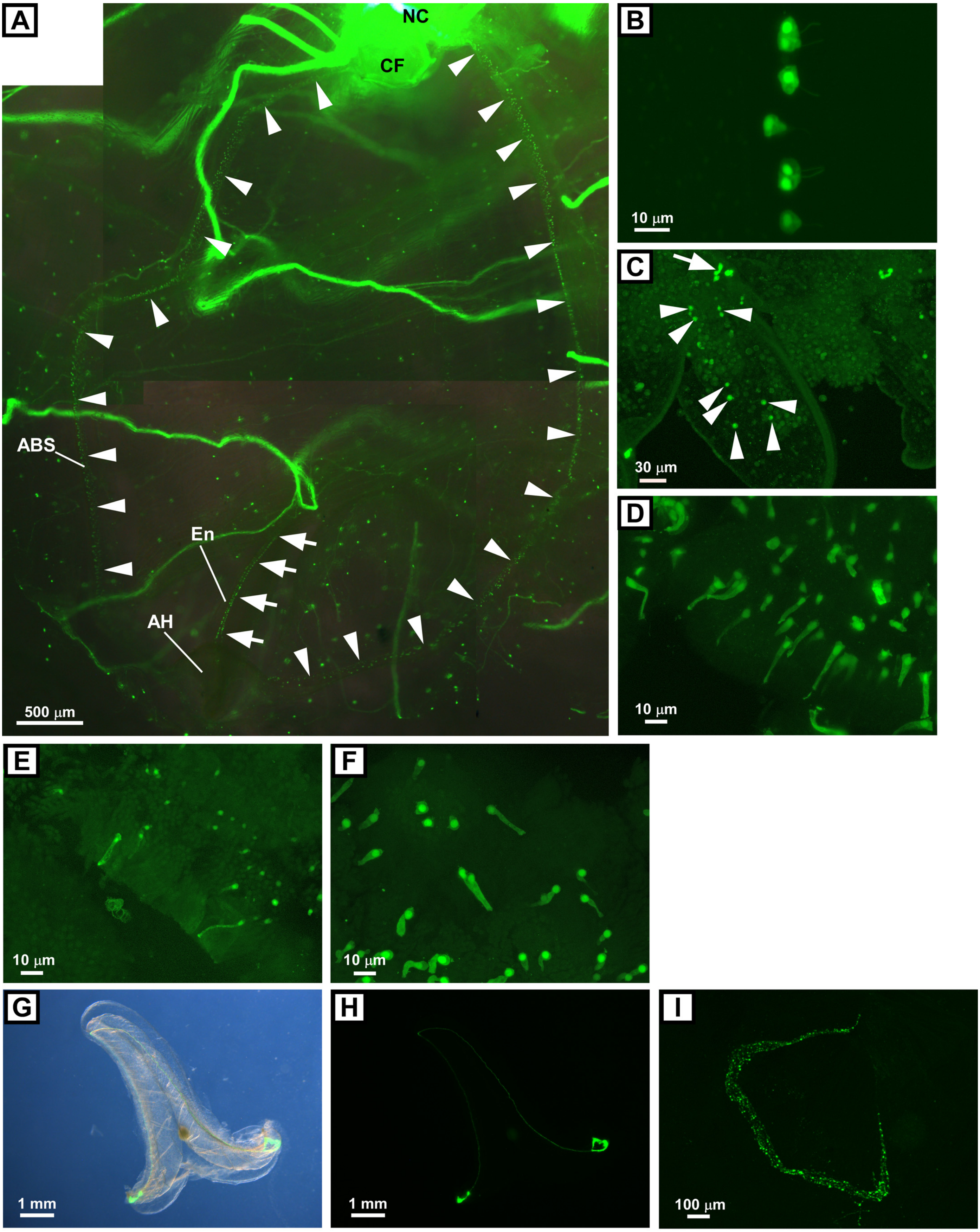
A
NC
CF
ABS
En
AH
500 µm
B
10 µm
C
30 µm
D
10 µm
E
10 µm
F
10 µm
G
1 mm
H
1 mm
I
100 µm

Fig. S1

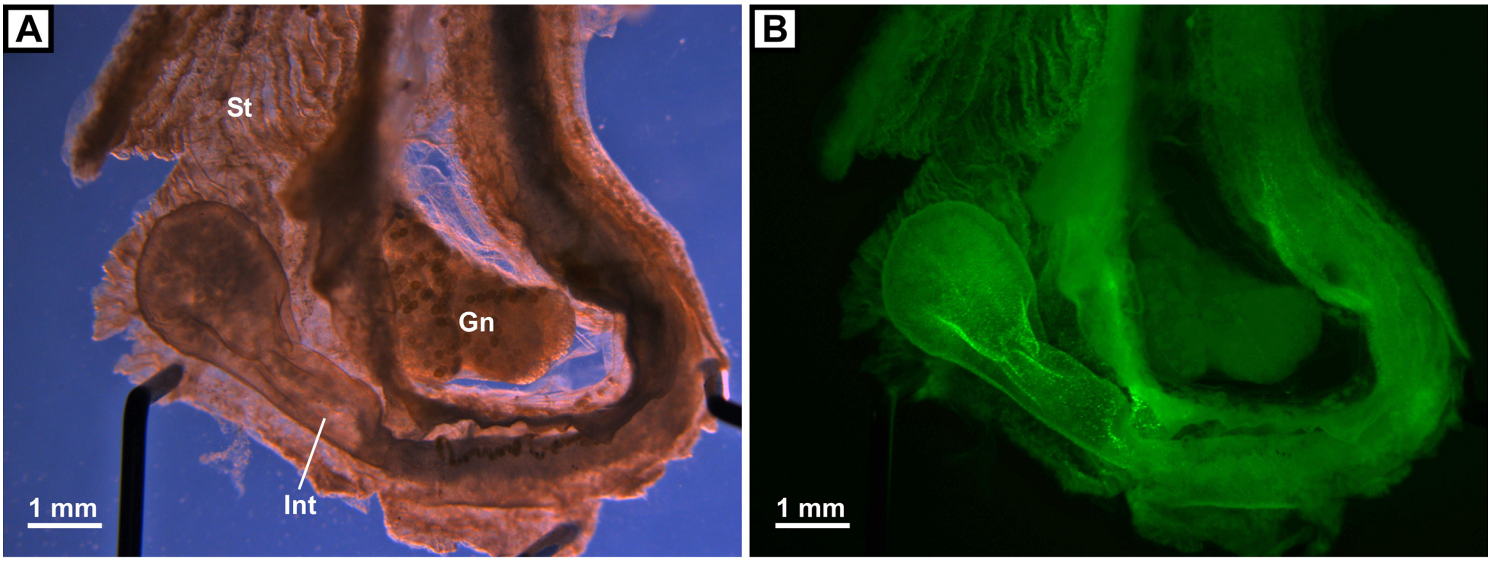


Fig. S2

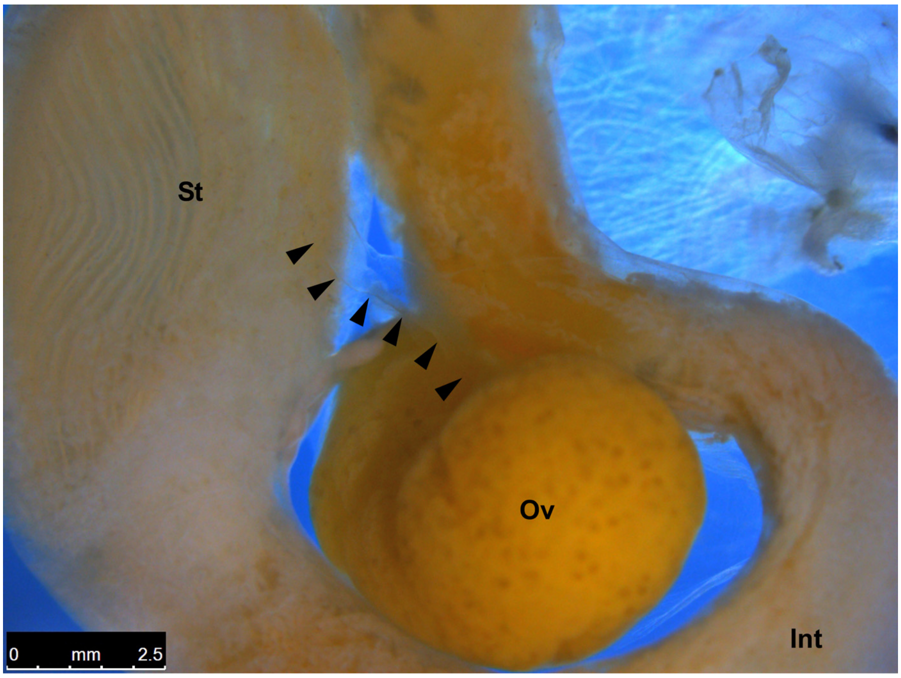


Fig. S3
